# Phosphorene quantum dot saturable absorbers for ultrafast fiber lasers

**DOI:** 10.1038/srep42357

**Published:** 2017-02-17

**Authors:** J. Du, M. Zhang, Z. Guo, J. Chen, X. Zhu, G. Hu, P. Peng, Z. Zheng, H. Zhang

**Affiliations:** 1 School of Electronic and Information Engineering, Beihang University, Beijing, 100191, China; 2 Key Laboratory for Micro-Nano Optoelectronic Devices of Ministry of Education, College of Physics and Microelectronic Science, Hunan University, Changsha 410082, China; 3 SZU-NUS Collaborative Innovation Centre for Optoelectronic Science & Technology, and Key Laboratory of Optoelectronic Devices and Systems of Ministry of Education and Guangdong Province, Shenzhen University, Shenzhen, China; 4 International Research Institute for Multidisciplinary Science, Beihang University, Beijing, 100191, China; 5 School of Mechanical Engineering and Automation, Beihang University, Beijing, 100191, China; 6 Collaborative Innovation Center of Geospatial Technology, Wuhan, 430079, China

## Abstract

We fabricate ultrasmall phosphorene quantum dots (PQDs) with an average size of 2.6 ± 0.9 nm using a liquid exfoliation method involving ultrasound probe sonication followed by bath sonication. By coupling the as-prepared PQDs with microfiber evanescent light field, the PQD-based saturable absorber (SA) device exhibits ultrafast nonlinear saturable absorption property, with an optical modulation depth of 8.1% at the telecommunication band. With the integration of the all-fiber PQD-based SA, a continuous-wave passively mode-locked erbium-doped (Er-doped) laser cavity delivers stable, self-starting pulses with a pulse duration of 0.88 ps and at the cavity repetition rate of 5.47 MHz. Our results contribute to the growing body of work studying the nonlinear optical properties of ultrasmall PQDs that present new opportunities of this two-dimensional (2D) nanomaterial for future ultrafast photonic technologies.

Ultrafast fiber laser sources are a mature technology that has become an essential tool facilitating a wide range of scientific and industrial applications^1^,^2^,^3^. This is driven by the continued development of saturable absorber (SA) technologies, along with low-loss new gain fiber medium, enabling versatile pulsed light sources. A SA acts as a passive optical switch in a laser cavity (i.e. reduced optical absorption with increased intensity of incident light) to produce ultrashort pulses using either mode-locking or Q-switching techniques^4^. The current dominant saturable absorber technologies, such as semiconducting saturable absorber mirror (SESAMs) and nonlinear polarization evolution (NPE) possess their own limitations, such as narrow operating bandwidth^2^, complex fabrication^5^ and packaging issues, and sensitive to environmental fluctuations. These limitations are driving research into the exploration of alternative materials with nonlinear optical properties for SA applications.

With advances in technology, the ability to manipulate the structure and composition at the nanoscale has opened the horizons to create new materials; of particular interests are 2D layered materials where reduced dimensionality leads to strong quantum confinement and novel physical phenomena^6^,^7^. Amongst the 2D layered material, graphene, consisting of mono- or few-layers of atoms, is one of the most successful examples and has been demonstrated to exhibit remarkable optical and electrical properties, including high optical nonlinear susceptibility, ultrafast carrier dynamics, broadband working wavelength range, in addition to robustness and environmental stability. This has led to the demonstration of numerous nonlinear optical effects of graphene, such as saturable absorption, Kerr nonlinearity and optical parametric processes, suggesting that this material could be a suitable platform for the development of photonic devices. In addition to graphene, semiconducting transition metal dichalcogenides (s-TMDs)^8^, another example of 2D layered material, have captured great research interest and been extensively exploited due to their diversity, and the distinct yet complementary physical properties to graphene they offer. S-TMDs (e.g. MoS_2_, MoSe_2_, WS_2_) are a family of ~40 different layered materials, where atoms within the layer are held together by strong covalent bond and the individual layers are stacked together by relatively weak van der Waals forces, which allow their exfoliation into single- and few-layer formats. Currently, a number of experimental and theoretical studies have been focused on exploring the applications of s-TMDs. For example, they have been demonstrated to Q-switch or mode-lock various laser cavities in a wide spectral range, covering from ~0.6 μm to 2 μm, due to their broadband nonlinear optical saturable absorption properties under strong illumination^8^,^9^,^10^,^11^,^12^. It has been reported that lasers using s-TMDs could operate in wavelengths below their fundamental bandgaps because of the sub-bandgap saturable absorption in these materials owing to the presence of edge states within the material bandgap that arises due to the boundaries of a finite crystal structure^7^,^13^,^14^. S-TMDs, however, are limited in a number of practical applications for optoelectronic devices as their intrinsic energy bandgap are in 1–2 eV^15^.

Recently, black phosphorus (BP) nanosheets^16^ have triggered rapid growing interest in both academic research and potential applications due to their unique structures and remarkable optoelectronic properties^12^,^17^,^18^. Similar to s-TMDs and other layered materials, the mechanical method has been successfully demonstrated to prepare single-and few-layer BP nanosheets^16^. Importantly, BP has a direct bandgap characteristics varying from 0.3 eV in bulk to 2 eV in monolayer and thus offers potential in bridging the gap between zero-bandgap graphene and large-bandgap s-TMDs. BP also offers the possibility of engineering its optical properties for desirable performances, such as saturable absorption and carrier dynamics. One such potential application exploiting saturable absorption property is the generation of short pulses by mode-locking or Q-switching in laser cavities. Table 1 summarizes nonlinear optical characteristics and applications in laser cavities using BP in the literature to date: Table 1(a) describes the properties of few-layer BP devices to mode-lock laser cavities and Table 1(b) presents the parameters for demonstrated BP-based SA devices for Q-switched lasers. The first demonstration of nonlinear optical properties of BP was performed by Chen *et al*.^17^, showing both Q-switched and mode-locked performance in Er-doped fiber laser cavities. The working wavelength of BP-based ultrafast lasers is then expended, covering from 1 μm to 3 μm^18^,^19^,^20^,^21^, manifesting its applicability as a new 2D broadband SA material.

Apart from the 2D layered structure, ultrasmall quantum dot, another form of nanomaterials, exhibiting unique properties owing to the quantum confinement and edge effects^22^,^23^, has been reported to possess prospective homogenous size and sizeable bandgap; and thus it offers new opportunities for tailoring optical properties for desirable photonic applications. For instance, it has been reported that graphene and MoS_2_ quantum dots possess promising properties and fascinating applications in photovoltaic devices^24^, optoelectronics^25^ and biological analysis^26^. Similarly, quantum dots of few-layer BP (or termed as phosphorene for single-layer), have been successively prepared using a facile top-down approach or liquid exfoliation method and find applications in fabricating memory devices and photothermal agents^16^,^27^. While the nonlinear response of BP quantum dots (BPQDs) has been reported at 800 nm by Xu *et al*.^28^, a comprehensive study of nonlinear optical properties of this material, in particular at 1.55 μm spectral region, and its versatile applications of ultrafast photonics is yet to be reported. For practical applications, the remarkable optical properties of the material at the optical communication band have received more attention due to the increasing interests in fabricating high-performance optical communication photonic devices. Therefore, it is of significant importance to investigate the nonlinear optical response of PQDs in the near infrared region, and the applicability as a potential SA device to produce ultrashort pulses in this spectral region.

Here, we fabricate the ultrasmall PQDs using a liquid exfoliation method combined with probe sonication and bath sonication, with an average size of 2.6 ± 0.9 nm. As for the applications of ultrafast photonics, the PQDs are deposited onto the microfiber for the integration of a SA. This SA device exhibits strong nonlinear response at 1.56 μm spectral region, indicating that the as-prepared device could be used as an ultrafast mode-locker for short pulse generation. Using this PQD-based SA device, we demonstrate the self-starting mode-locked pulses generated from an Er-doped fiber laser to underscore its applicability as a broadband SA material.

## Results and Discussion

### Sample preparation and characterization

The PQDs are prepared by the liquid exfoliation method, an approach previously demonstrated for other 1D and 2D materials^13^,^14^,^29^,^30^ (see Methods), and involves ultrasound probe sonication followed by bath sonication of ground powder of bulk according to our previous work^31^.

**Figure 1:**
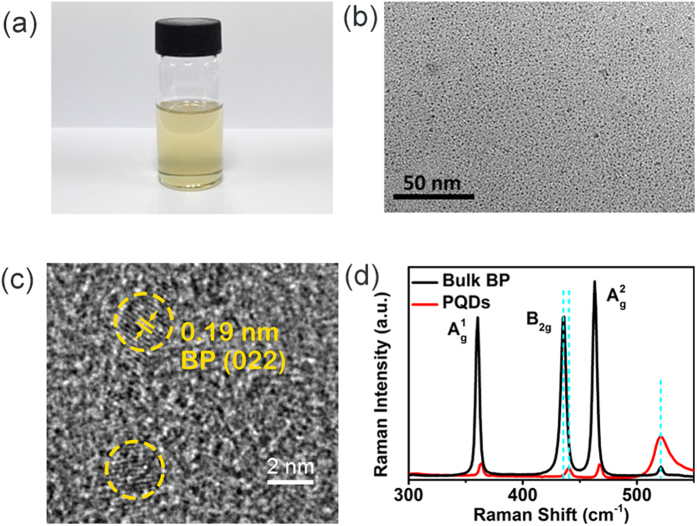
Optical images of BP liquid dispersion (**a**) photograph of the undiluted dispersion, (**b**) TEM image, (**c**) HRTEM image, and (**d**) Raman spectra of PQDs.

The photograph of a cuvette of the undiluted dispersion is shown in Fig. 1(a). The transmission electron microscopy (TEM) image of the as-synthesized PQDs appears as uniform dots with a diameter of 2.6 ± 0.9 nm [Fig. 1(b)], similar to that reported in the previous literature^16^,^27^. We then characterized the dispersion via high resolution transmission electron microscopy (HRTEM). As shown in Fig. 1(c), the distance between the adjacent hexagonal lattice fringes is measured to be 0.19 nm [shown in Fig. 1(c)], which is consistent with the lattice spaces of the (022) plane. Raman spectra show all three vibration peaks of the PQDs at 364.3, 440.5, and 467.1 cm^−1^ shifted to slightly larger wavenumbers compared with those of bulk BP [Fig. 1(d)]. Such blue shifts (3.8 cm^−1^, 4.3 cm^−1^, 4.2 cm^−1^, respectively), in comparison with the shifts of BPQDs with different layer thickness reported in ref. 32, indicate that our PQDs are 1–2 layer thick. The 440.5 cm^−1^ and 467.1 cm^−1^ peaks belong to the B_2g_ and A_g_^2^ vibration modes of different crystalline orientations within the layer plane. The relatively high intensity of these two peaks indicates that the as-synthesized PQDs remain crystalline structure after the exfoliation process as confirmed by HRTEM observation [as shown in Fig. 1(c)].

To develop practical and flexible saturable absorber devices for laser applications, the as-prepared PQDs can be integrated using a number of optically-compatible strategies^7^, such as directly deposited on fiber ferrules using optical deposition method^33^,^34^, embedded in transparent polymer composite^13^,^35^ and transferred to the tip of an optical device (e.g. fiber or reflected mirror)^36^,^37^ as a post-processing step. In our experiment, the PQD-based SA that used for ultrashort pulse generation in the laser cavity, is realized by the nonlinear interaction of the processed material with the evanescent field of light in a microfiber. The microfiber is prepared by polishing a single-mode fiber after holding with an arcuate block. An optical power meter is used to monitor the insertion loss which indicates the space between the fiber core and polished surface. The waist diameter is ~10 μm and the length is ~10 mm. The insertion loss of the integrated microfiber is measured to be 0.4 dB with a continuous laser centered at 1554.5 nm. The PQD dispersion is deposited on the microfiber by using an optical deposition method^38^. The PQD solution is dropped on the cross section of the microfiber which is fixed on a quartz plate. A 980 nm continuous wave laser with an optical power of 60 mW is then coupled into the microfiber and the optical deposition process starts. The light propagating through the microfiber device is collected by an optical power meter to evaluate the deposition depth in real time. This approach is similar to other layered material based SA devices via evanescent field mediating the strength of the light-matter interaction on tapered or D-shaped fibers^34^.

**Figure 2:**
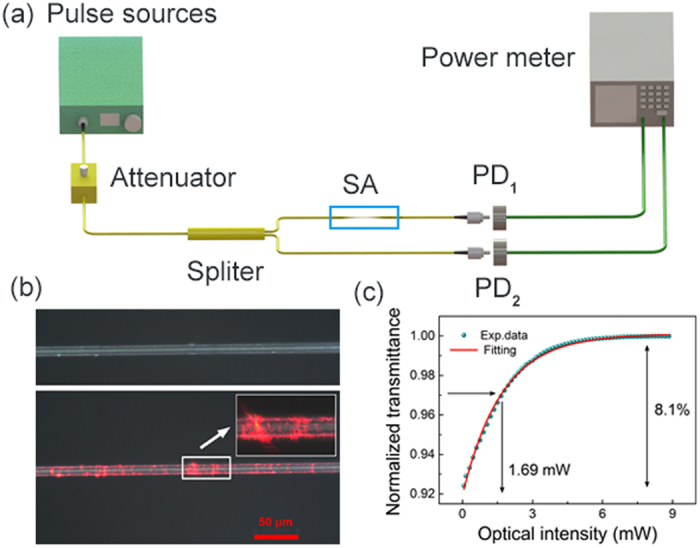
(**a**) The setup of a balanced twin-detector measurement, (**b**) Photograph of the microfiber deposited with PQDs, the upward and downward images show the integrated microfiber devices before and after injecting a 650 nm laser source, and the inset shows a zoom-in image of the device, (**c**) saturable absorption property of the PQD-SA device.

The microscope images of the microfiber-based PQD-SA device are shown in Fig. 2(b). The upward image shows the microfiber coated with PQDs at a magnification of 500 times; the downward image and the inset, at a magnification of 500 and 1000 fold, respectively, presents the light-matter nonlinear interaction in the microfiber device after injecting a 650 nm He-Ne laser source. The nonlinear optical absorption of the integrated PQD-SA is characterized using a power-dependent transmission technique based on a balanced twin-detector measurement system. As shown in Fig. 2(a), a home-made ultrashort fiber laser source operating at 1560 nm is used as the pump light (500 fs pulse duration, 20 MHz repetition rate), splitted using a 50:50 fused fiber coupler, the latter enabling monitoring of power as a reference. By continuously adjusting the attenuator, the transmitted power is recorded as a function of incident optical power on the integrated microfiber SA device. A typical dataset from a single balanced twin-detector measurement, shown in Fig. 2(c), can be well fitted with a two-level SA model^34^. From the fit, the saturable average power and normalized modulation depth of the device are extracted to be 1.69 mW and 8.1%. Thus, the PQD-SA shows strong saturable absorption property illustrating potential to be used for short pulse generation in a laser system.

### Demonstration of mode-locking a fiber laser using PQD-SA

**Figure 3:**
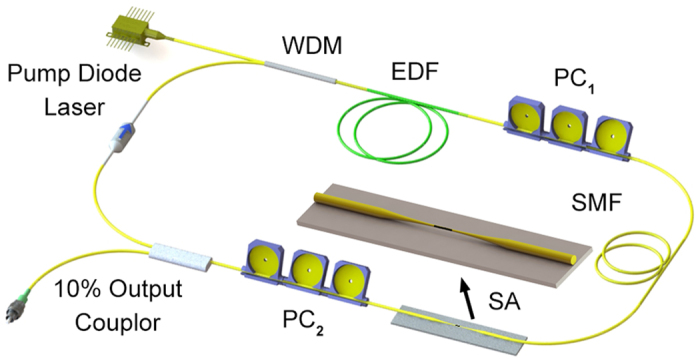
The schematic of ultrafast Er-doped fiber laser using a microfiber-based PQD-SA and an enlarged configuration of the SA device.

The demonstrated saturable absorption of the microfiber-based PQD-SA at 1560 nm spectral region indicates that the device could be used to introduce a self-amplitude modulation of a fiber laser cavity. This could in turn be exploited to generate a regular train of mode-locked pulses in this spectral region. We developed an Er-doped fiber laser consisting of single-mode all-fiber integrated components for an alignment-free and compact system, as shown in Fig. 3. The fiber amplifier consists of a length of 0.7 m single-mode Er-doped active fiber (LIEKKI Er80-8/125) with a group velocity dispersion (GVD) of −22.6 ps^2^ km^−1^, co-pumped by a 980 nm pump laser diode. In addition to the fiber amplifier, the cavity includes a polarization-independent optical isolator (PI-ISO) to ensure unidirectional propagation, 10:90 fused fiber output coupler for both spectral and temporal diagnostics, and polarization controllers (PC_1_ and PC_2_) to enable a thorough and continuous adjustment of the net cavity birefringence but is not fundamental to the mode-locking action. The total cavity length is ~37.8 m and the net cavity dispersion β_2_ is calculated to be ~−0.6 ps^2^, ensured the laser operating in the average-soliton regime^39^.

**Figure 4:**
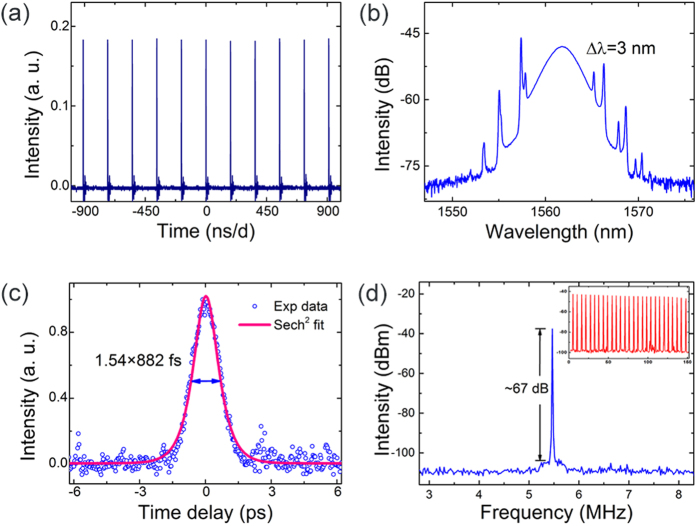
Mode-locking performance of the Er-doped fiber laser using a microfiber-based PQD-SA device: (**a**) output pulse train, with a spacing of 183.2 ns; (**b**) measured optical spectrum; (**c**) autocorrelation of the output pulses, with a deconvolved duration of 882 fs, (**d**) radio frequency spectra of fundamental frequency and the inset of higher cavity harmonics.

**Figure 5:**
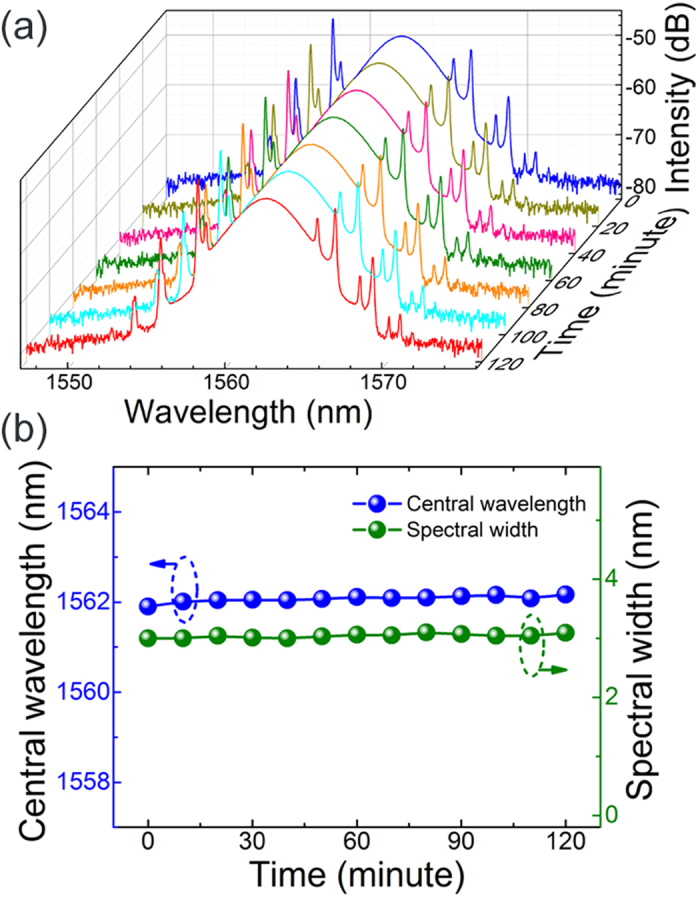
(**a**) Measured optical spectra of the mode-locking performance at 20 mins interval; (**b**) the drift of the central wavelengths and the 3 dB spectral widths.

Self-starting mode-locking is observed at the fundamental repetition frequency of the cavity of 5.47 MHz [Fig. 4(a)], with 24.7 pJ single pulse energy. Figure 4(b and c) show the spectral and temporal profile of the output pulses. The spectrum is centered at 1561.7 nm, with a full width at half maximum (FWHM) of 3 nm [Fig. 4(b)]. The corresponding pulse duration, measured using an intensity autocorrelator, is 882 fs (deconvolved), well fitted with a sech^2^ pulse shape and plotted in Fig. 4(c). The time-bandwidth product is calculated to be 0.325, close to the Fourier transform limit of a sech^2^ pulse^40^. The radio frequency (RF) spectra, which could be used to infer the laser stability^41^, are shown in Fig. 4(d). The fundamental frequency shows a high signal-to-background extinction ratio of ~67 dB, indicating low-amplitude fluctuations, and stable mode-locking operation performance. The inset of Fig. 4(d) shows higher cavity harmonics, recorded on a span of 150 MHz, without any noticeable sign of Q-switching instabilities, implying good pulse-train stability. To further evaluate the operating stability of the microfiber-based PQD-SA device and mode-locking performance of the fiber laser, we recorded the optical spectra of the laser every 20 mins over 2 hours, presented in Fig. 5. No evident variation of both central wavelength (with standard deviation of 0.05 nm) and spectral bandwidth (with standard deviation of 0.03 nm) could be observed, suggesting that the mode-locking operation possesses a reasonably good operating performance.

The net cavity GVD is anomalous, facilitating soliton pulse shaping through the interplay of GVD and self-phase modulation (SPM). This is confirmed by the observation of narrow peaks superimposed on the soliton-pulse spectrum– arising from resonances between the soliton and dispersive wave components emitted after soliton perturbations. However, despite the laser consisting of non-polarization discrimination components, the position of one set of the spectral sidebands shifts when deliberately adjusts the intracavity PC while the other set remains unchanged which indicates the formation of vector soliton in the cavity. To clearly identify the difference of the spectral sidebands, the laser output is splitted into two orthogonal polarization components by using a polarization controller that used to balance the fiber pigtail induced linear polarization rotation and followed by a fiber pigtailed external cavity polarization beam splitter.

**Figure 6:**
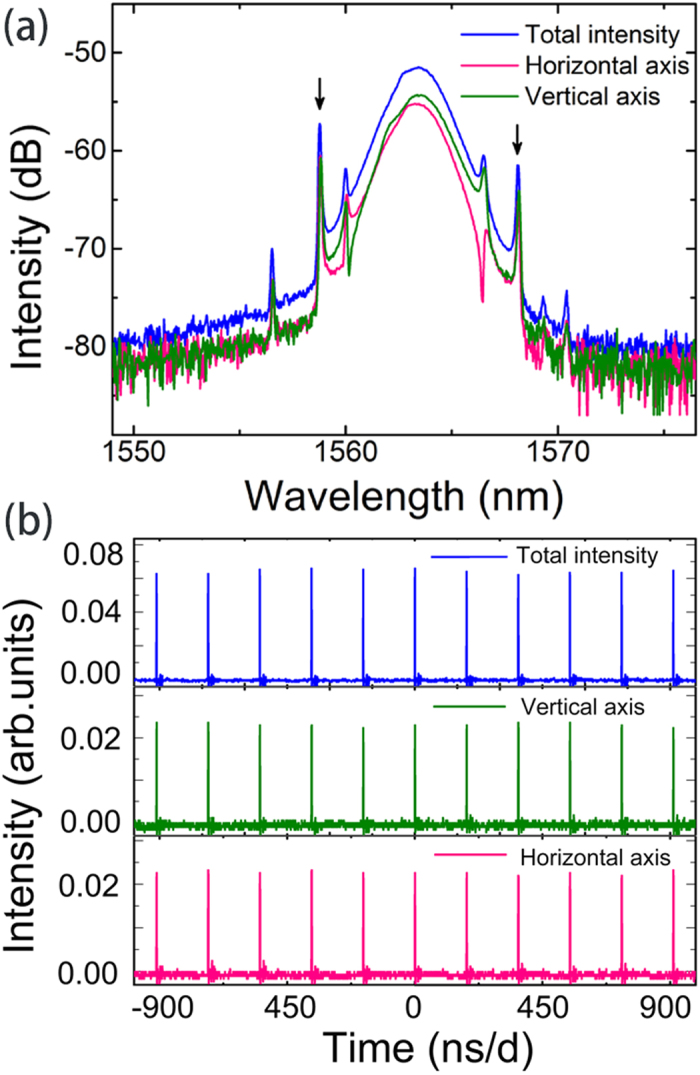
Vector solitons emission of the fiber laser, blue line: laser emission characterization without passing through a polarizer, green and pink lines: two orthogonal polarization components resolved with an external cavity: (**a**) soliton spectra, (**b**) oscilloscope traces of the pulse train.

The polarization resolved spectra of the solitons were measured simultaneously, and plotted in Fig. 6(a) with a green line and pink line showing the vertical and horizontal axis, respectively. It is clear to note that, in the polarization resolved spectra, one set of the spectral sidebands displays either as a spectral peak or spectral dip, between the two orthogonal polarization components while the solitonic sidebands always exhibit as spectral peaks (labeled with arrows)^42^. Such peak-dip relationship indicates the existence of coherent energy change between the two soliton components^43^. While previous literature have reported four-wave mixing between the two orthogonal polarization components of light in weakly birefringent SMFs experimentally and numerically^44^,^45^ which could cause polarization instability, we did not observe such phenomenon in our cavity as the formation of a stable soliton in a laser cavity is required to satisfy the gain-loss balance condition which is different from the case of light propagation in standard SMFs that treated as a conservation system^46^. Figure 6(b) is the measured oscilloscope trace of the soliton pulses. All pulses in the cavity have the same height, indicating that polarization locked vector solitons were formed^47^,^48^,^49^. This type of vector soliton is under the intra-cavity birefringence with a locked group velocity and phase velocity. The nonlinear birefringence induced by self-phase modulation, cross-phase modulation and four-wave mixing coherent energy coupling compensates the intrinsic birefringence, and as such solitons could maintain their own polarization states.

## Discussion

Following stable mode-locking results obtaining by using the microfiber-based PQD-SA device in the Er-doped fiber laser, the same experiment was conducted with the same microfiber device after removing the deposited PQD material. No mode-locking state (or any other pulsating performance) could be observed at any power level or polarization controller position, confirming that the saturable absorption arises from the PQDs at 1560 nm spectral region.

As discussed above, the bandgap of this conceptually new layered material is strongly layer-dependent, spanning from visible even to the mid-infrared region, perfectly bridging the gap between zero-bandgap graphene and s-TMDs, which suggests that this material could play a role in enabling low-cost and flexible saturable absorber devices for integrated pulsed fiber lasers. While the strong nonlinear saturable absorption and nonlinear behavior of PQD-SA device is verified by the intensity-dependent absorption measurement and demonstration of a mode-locked fiber laser at 1561.7 nm, the stability of BP-based devices remain a critical problem for practical applications as they might easily encounter oxidation if being exposed in air without particular external protections. We experimentally found that PQD dispersion in NMP solvent possesses a good stability for half a year without degradation. More importantly, such problem could be overcome by the introduction of BPs with other structures, such as organic polymer that can protect the oxidation form the air/water or other layered materials that exhibit stable in air^50^. Despite a very simple cavity consisting of non-polarization maintaining fiber, the laser emits high-quality pulses (relatively small fluctuation of the spectral profile over 2 hours), indicating that this material could be a promising 2D SA candidate for ultrafast optics, and its unique optical properties promise to shape the future of photonic technologies^7^,^51^.

## Conclusions

In summary, the ultrasmall PQD material has been fabricated by the liquid phase exfoliation in NMP, involving probe sonication and bath sonication. As a proof-of-concept demonstration, the as-prepared PQDs have been deposited onto the microfiber to form a SA device, exhibiting strong nonlinear saturable absorption properties, for ultrafast laser cavities via evanescent field interaction. Using this SA, we have developed a self-starting mode-locked Er-doped fiber laser for ultrashort pulse generation, in addition to the observation of polarization locked vector solitons. Such results extend our understanding of BP to a wider class of 2D layered materials with regard to their potential for ultrafast photonic applications, which could leverage benefit in short-pulse fiber laser technology.

## Methods

### Preparation of few-layer PQDs

The PQDs are fabricated using a liquid exfoliation technique in N-methyl-2-pyrrolidone (NMP) solvent involving an ultrasound probe sonication followed by the bath sonication of ground powders of bulk BP according to our previous work^26^. Briefly, 0.5 mg BP powder purchased from *Smart Elements* is added into 1 mL of NMP in a mortar and the mixture is grounded for 20 mins. The mixture is then transferred into a 15 mL glass vial with the addition of another 3 mL NMP. After the glass vial is carefully sealed, the vial is put in an ice-bath sonicator and sonicated at the power of 200 W for 3 h. After the exfoliating process, the suspension is centrifuged at 7000 rpm to remove undefoliated grounded bulk BP particles. The supernatant is centrifuged at 12,000 rpm for 20 mins in order to separate the as-synthesized PQDs. The precipitate is washed and re-dispersed in the NMP solution for further applications.

### Balanced twin-detector measurement

The balanced twin-detector measurement is based on a home-made tunable femtosecond pulses, with 500 fs pulse duration, 20 MHz cavity repetition rate, tunable spectral profile from 1530 to 1565 nm. Specifically, we have chosen an excitation wavelength of 1560 nm to study the nonlinear optical saturable absorption of PQD in the telecommunication band. An electrical controlled attenuator is employed to precisely control the incident optical power. During the measurement, we did not observe any laser damages either caused by the high intensity or thermal effects with the maximum power of 9 mW.

## Additional Information

**How to cite this article**: Du, J. *et al*. Phosphorene quantum dot saturable absorbers for ultrafast fiber lasers. *Sci. Rep.* **7**, 42357; doi: 10.1038/srep42357 (2017).

**Publisher's note:** Springer Nature remains neutral with regard to jurisdictional claims in published maps and institutional affiliations.

## Figures and Tables

**Table 1:** Short pulsed Lasers with BP Nano Flake SAs.

(a) Layered BP Flakes Integrated to Form SA Device for Mode-locked Lasers									
Fabrication Method	Integration Platform	Layers in BP Flakes	Nonlinear Characterization		Laser Type	Laser Properties			Ref.
			I_s_ (MW/cm^2^)	α_s_ (%)		λ (nm)	t	TBP	
ME	Fiber facet	15	6.55	8.1	Er: Fiber	1571.45	946 fs	0.328	17
LPE	Microfiber	1–3	~4.5 mW	6.91	Er: Fiber	1532–1570	940 fs	0.38	31
LPE	Quartz	~8	1.35	7.5	Nd:YVO_4_	1064.1	6.1 ps	0.464	18
ME	Fiber facet	~500^#^	—	0.6–4.6	Er: Fiber	1560.5	272 fs	0.34	19
ME	Fiber facet	~500	—	4.1	Tm: Fiber	1910	739 fs	0.352	33
LPE	Side-polished Fiber	~20	~12.5	3.31	Er: Fiber	1558.14	2.18 ps	0.336	29
ME	Fiber facet	~33–1833^#^	—	50–90	Er: Fiber	1558.7	~786 fs	~0.6	52
LPE	Microfiber	~100	—	9.8	Tm/Ho: Fiber	1880–1940	1.58 ps	0.486	20
ME	Gold-coated mirror	~238	9 μJ/cm^2^	19	Er: ZBLAN	2783	42 ps	4.5	21
LPE	Fiber facet	~3–41^#^	3.41	4.48	Er: Fiber	1568.19	117.6 ns	—	53
ME	Fiber facet	5–8	0.35	8	Yb: Fiber	1085.5	7.54 ps	0.441	54
**(b) Layered BP Flakes Integrated to Form SA Device for Q-switched Lasers**									
**Fabrication Method**	**Integration Platform**	**Layers in BP Flakes**	**Nonlinear Characterization**		**Laser Type**	**Laser Properties**			**Ref.**
			**I**_**s**_ **(MW/cm**^**2**^)	**α**_**s**_ **(%)**		**λ (nm)**	**t**_**min**_	**E**_**max**_	
ME	Fiber facet	25	10.74	18.55	Er: Fiber	1562.87	10.32 μs	94.3 nJ	17
LPE	Gold-coated mirror	~8–33^#^	9 μJ /cm^2^	15	Er: ZBLAN	2779	1.18 μs	7.7 μJ	55
ME	Fiber facet	~33–1833^#^	—	50–90	Er: Fiber	1532.5	~3.1 μs	~18.6 nJ	52
LPE	Reflector	5–15	—	—	Yb: CYA	1046	~620 ns	~325.7 nJ	36
LPE	PVP composite	~6–41^#^	—	—	Er: Fiber	1561.9	2.96 μs	194 nJ	56
ME	Quartz	40–50	—	—	Pr: GdLiF_4_	639	189 ns	104 nJ	57
ME	Quartz	40–50	6.14 GW/cm^2^	35.48	Nd: GdVO_4_	1.06 μm	495 ns	70.4 nJ	57
ME	Quartz	40–50	—	—	Tm: Ho: YGG	2.1 μm	636 ns	221 nJ	57
LPE	Quartz	~10	0.96	10.7	Cr: ZnSe	2411	189 ns	205 μJ	58
LPE	Fiber facet	~38	1.1	24	Tm/Ho: Fiber	1912	731 ns	632.4 nJ	59
ME	Gold-coated mirror	—	20 μJ /cm^2^	5	Tm:YAG	2009	2.9 μs	3.32 μJ	60
ME	Quartz	60–80	6.93 GW/cm^2^	13.8	Yb: ScBO_3_	1063.6	495.5 ns	1.4 μJ	61
LPE	Gold-coated mirror	38^#^	—	—	Yb: LuYAG	1029	1.73 μs	0.09 μJ	62
LPE	Gold-coated mirror	38^#^	1.15 μJ /cm^2^	7.8	Tm: CaYAlO_4_	1930	3.1 μs	0.68 μJ	62
LPE	Gold-coated mirror	38^#^	—	—	Er: Y_2_O_3_	2.72 μm	4.47 μs	0.48 μJ	62
MP	Side-polished Fiber	<16666^#^	—	—	Er: Fiber	1550	9.35 μs	28.3 nJ	63
MP	Side-polished Fiber	<16666^#^	—	—	Tm/Ho: Fiber	1832–1935	2.53 μs	276 nJ	63

Nonlinear optical characteristics and applications in laser cavities using BP, (a) for mode-locked lasers and (b) for Q-switched lasers. Where BP-based SAs were applied to Q-switched lasers, we quote the minimum value of pulse duration (t_min_) and maximum value of pulse energy (E_max_), respectively. ME, mechanical exfoliation; LPE, liquid phase exfoliation; MP, mechanically polishing; I_s_, saturating intensity; α_s_, modulation depth; λ, operating wavelength; t, pulse duration; TBP, time-bandwidth product. ^#^Indicates that layers in flakes were not given (using the thickness provided in literature for the calculation) so has been estimated instead by adopting the theoretical value of 0.6 nm for single-layer phosphorus in ref. 64.
